# Descriptive study on esketamine and ketamine treatment in real-world patients with a diagnosis of resistant depression

**DOI:** 10.1192/j.eurpsy.2025.868

**Published:** 2025-08-26

**Authors:** E. A. Ródenas Vera, S. Sabater, P. Alvarez, S. Oller, I. Riedel

**Affiliations:** 1 psychiatry department; 2 Hospital del Mar, Barcelona, Spain; 3 Psychatry, Hospital del Mar, Barcelona, Spain

## Abstract

**Introduction:**

Depressive disorder is one of the health problems that carries the greatest burden of morbidity, with high prevalence and impact on people’s quality of life. It also affects the family environment and contributes to the social and economic burden on health systems (World Health Organization, depression, 2023). Currently, the treatment of depression has limitations and there is a high frequency of patients who do not respond despite multiple trials of antidepressants. Up to two-thirds of patients diagnosed with depression do not achieve remission despite treatment, and 30% of patients are considered treatment-resistant, defined as a minimum of two failures to previous treatments, in adequate doses and duration (Gaynes et al., 2019). Recent innovations in the management provide promising opportunities to improve the symptomatology of these patients. New drugs such as ketamine and esketamine, which have glutamatergic neuromodulatory properties, are used under supervision for the treatment of patients with treatment-resistant depression (Vasiliu, 2023).

**Objectives:**

The aim is to describe a sample of real-world patients with a diagnosis of resistant depression referred to esketamine/ketamine treatment. The individuals were being followed by psychiatrists of a public hospital in the city of Barcelona and were selected to start treatment indicated by the refractoriness and severity of the episode.

**Methods:**

We used a database that collected multiple sociodemographic, clinical and treatment variables of 32 patients with refractory depression who were referred to treatment with esketamine/ketamine. SPSS software was used for data processing. All the patients in the group were followed up by psychiatry in a public hospital in the city of Barcelona during the period from July 2015 to September 2024.

**Results:**

Of the 32 patients evaluated, 11 were male (34.4%) and 21 were female (65.6%). The mean age at the time of receiving ketamine/ketamine treatment was 53 years with a standard deviation of 10.7. Nearly 60% had a comorbid psychiatric diagnosis. Twenty-eight percent had undergone electroconvulsive therapy. The mean number of previous episodes was 3.72 with a median of 2.5. Regarding the response to treatment we found that it was partial in 15 patients (46.9%) and complete remission could be obtained in 7 patients (21.9%), with no response to treatment in 10 of them (31.2%). In 5 patients the response was considered a late response.

**Conclusions:**

In most of the patients a partial improvement was assessed as evidenced by a reduction in the Montgomery-Asberg Depression Rating Scale (MADRS). Few cases obtained a complete remission with treatment.

As limitations to the results, we can refer to the small sample size. However, we consider that the severity and chronicity of the episodes make the description of the response in a real world group seem of interest for future studies.

**Disclosure of Interest:**

None Declared

